# Effectiveness of peer counseling and membership in breastfeeding support groups in promoting optimal breastfeeding behaviors in the Philippines

**DOI:** 10.1186/s13006-021-00400-5

**Published:** 2021-07-12

**Authors:** Ofelia P. Saniel, Veincent Christian F. Pepito, Arianna Maever L. Amit

**Affiliations:** 1 College of Public Health, University of the Philippines Manila, 625 Pedro Gil St., Ermita, Manila, Philippines; 2 Symmetrix Research Consultancy Company, Manila, Philippines; 3 School of Medicine and Public Health, Ateneo de Manila University, Ortigas Ave., Pasig City, Philippines

**Keywords:** Millennium Development Goals, Breastfeeding support group, Peer counselor, Early initiation of breastfeeding, Exclusive breastfeeding, Breastfeeding, Pediatrics, Philippines

## Abstract

**Background:**

The prevalence of early initiation of breastfeeding and exclusive breastfeeding (EBF) at 6 months remain low in the Philippines. To help meet the 90% early initiation of breastfeeding target and to improve infant and young child feeding practices in the Philippines, the Millennium Development Goals - Fund 2030 Joint Programme (JP) on Ensuring Food Security and Nutrition for Children 0–24 months old was implemented. We aimed to determine the effectiveness of visits by peer counselors during pregnancy and after delivery, and membership in breastfeeding support groups in promoting these optimal breastfeeding practices.

**Methods:**

We used data from the Endline Survey of the JP to study the effects of prenatal and postnatal peer counselor visits, and membership in breastfeeding support groups, and their possible interactions with initiation of breastfeeding within 1 hour of birth among children aged 0 to 24 months and EBF at 6 months among children aged 6 to 24 months, while adjusting for confounding. We used logistic regression methods for survey data to assess these associations.

**Results:**

Of the 2343 mother-infant pairs, only 1500 (63.1%) practiced early initiation of breastfeeding. Of the 1865 children aged 6 months or older, only 621 (34.7%) were exclusively breastfed at 6 months. After adjusting for confounding variables, there was no strong evidence that peer counselor visits were associated with early initiation or EBF at 6 months. However, members of breastfeeding support groups had 1.49 times higher odds of early initiation of breastfeeding (95% CI [Confidence Interval] 1.12, 1.98) and 1.65 times higher odds of EBF (95% CI 1.20, 2.24) compared to those who were not members of breastfeeding support groups. There was no interaction between the different exposure variables and early initiation and EBF at 6 months.

**Conclusions:**

Our findings suggest breastfeeding support groups may be institutionalized to promote both early initiation of breastfeeding and EBF in the Philippines, while the role of peer counselors in promoting optimal breastfeeding behaviors should be further reviewed. Our suggestion to integrate non-healthcare professionals to promote early initiation of breastfeeding and EBF could be tested in future intervention studies.

**Supplementary Information:**

The online version contains supplementary material available at 10.1186/s13006-021-00400-5.

## Background

Early initiation of breastfeeding, defined as breastfeeding within 1 h of birth, and exclusive breastfeeding (EBF), defined as giving the infant breastmilk only, without supplementary food or water or medicines, during the first 6 months of life, are optimal breastfeeding behaviors that improve child health and survival [1–5]. Early initiation of breastfeeding also stimulates the production of breastmilk, provides antibody protection, and reduces postpartum hemorrhage [6]. Further, it is linked with successful practice of other optimal breastfeeding behaviors, such as EBF for 6 months after birth, and continued breastfeeding for at least 2 years with complementary feeding after 6 months [5, 7, 8]. Longer duration of EBF significantly improves motor and cognitive development [9, 10], and prevents diarrhea, acute respiratory infections, and fever among infants. Exclusive breastfeeding also reduces the burden of undernutrition in the community [11]. In addition to the direct benefits of optimal breastfeeding behaviors on infants, breastfeeding also benefits mothers. In the short term, breastfeeding reduces maternal bleeding and stress after delivery, facilitates positive metabolic changes and postpartum weight loss, and delays ovulation. In the long term, breastfeeding increases postpartum weight loss, decreases visceral adiposity, and reduces risk of cardiovascular diseases, diabetes, and breast and ovarian cancers [12].

Despite the benefits of early initiation of breastfeeding and EBF, their prevalence is low in many countries, including the Philippines. The overall prevalence of early initiation of breastfeeding was only 57.6%, in 24 countries included in the World Health Organization (WHO) Global Survey on Maternal and Perinatal Health in 2004 to 2008. The same survey also reported that the Philippines ranked among the lowest, with an early initiation of breastfeeding prevalence of only 39.9% [6]. Meanwhile, there has been little progress in improving the practice of exclusive breastfeeding. As of 2015, only 40% of children worldwide were exclusively breastfed [13]. The Philippine National Demographic and Health Surveys reported that the prevalence of early initiation of breastfeeding within the first hour of life in the country was 54.0% in 2003 and 53.5% in 2008, while the prevalence of EBF, defined as the proportion of infants under 6 months of age who were given only breastmilk, were 33.5% and 34.0%, respectively [8]. However, the 2013 Philippine National Demographic and Health Survey reported that the prevalence of early initiation of breastfeeding in the country further decreased to 49.0% [14]. These figures are far from the targeted 90% prevalence of early initiation of breastfeeding and the 70% prevalence of EBF under 6 months of age set by the Philippine Department of Health (DOH) by 2016 [8].

To help meet these targets and to improve infant and young child feeding (IYCF) practices in the Philippines, the WHO, the United Nations Children’s Fund (UNICEF), the Food and Agriculture Organization (FAO), the International Labor Organization (ILO), and the World Food Program (WFP), in collaboration with the DOH, launched in 2009 the Millennium Development Goals - Fund 2030 Joint Programme (MDG-F JP) on Ensuring Food Security and Nutrition for Children 0–24 months old in the Philippines. The overall aim of the Programme was to accelerate the attainment of Millennium Development Goals (MDGs) 1 and 4, which focused on reducing undernutrition among children and decreasing child mortality, respectively. Other key outcomes of the JP were increased prevalence of EBF by 20% annually, reduced prevalence of undernutrition by 3%, and improved capacities of national and local government units and stakeholders to formulate, promote, and implement IYCF policies and programs. To attain these outcomes, several intervention projects were implemented including the engagement and training of volunteer breastfeeding peer counselors and the establishment of breastfeeding support groups to encourage mothers to practice optimal breastfeeding during pregnancy and after childbirth. After the JP implementation, an external agency conducted a cross-sectional study between 2012 and 2013 to assess the outcomes and impact of the Programme. A 0.7% reduction in the prevalence of undernutrition and 7.7% increase in the prevalence of EBF were recorded after the implementation of the JP interventions; however, both figures were far from the JP targets. The same cross-sectional study also reported that the prevalence of early initiation of breastfeeding was 62.8% [15, 16]. While an impact evaluation study has been conducted to assess the overall effectiveness of the JP, the effectiveness of component projects like the JP-trained peer counselors and the breastfeeding support groups in promoting early initiation of breastfeeding and EBF among target mothers is unknown.

Previous studies showed that the determinants of early initiation of breastfeeding include knowledge about optimal feeding practices [17, 18], mother’s age [17, 19, 20], mother’s and partner’s employment status [21–23], and exposure to breastfeeding information during pregnancy [17, 24]. Other factors associated with early initiation of breastfeeding are place of delivery, birth attendant, mode of delivery [18, 20, 23], and knowledge about breastfeeding [18, 25]. A previous study in the Philippines reported that after adjusting for confounding variables, first breastfeed in infants from mistimed pregnancies was much later than infants from planned pregnancies among households with low socioeconomic status [26]. A systematic review on the topic confirmed the role of the aforementioned determinants on early initiation of breastfeeding. Other factors such as place of residence, educational attainment of the parents, socio-economic status, and infant’s sex were reported to be important determinants as well [27, 28]. The determinants of EBF, on the other hand, include mother’s marital status, mode of delivery [29, 30], economic independence, maternal age, number of children, socio-economic status, maternal HIV status, and birthweight of infant [31, 32]. Having antenatal care, maternal education, and maternal employment were associated with lower odds of exclusive breastfeeding [32]. The same set of variables was also found to be associated with EBF in two systematic reviews, in addition to place of residence, religion, rooming-in, pre-lacteal feeding, advice from relatives and peer-pressure, infant’s sex, maternal smoking, and exposure to advertisements about breast milk substitutes [27, 33].

Although some studies on the determinants of early initiation of breastfeeding and breastfeeding duration have been conducted in the Philippines [26, 34], this is the first study, in our knowledge, to explore the effectiveness of specific interventions to promote early initiation of breastfeeding and EBF in the country. This paper’s aim was to examine the independent association of peer counseling and membership in breastfeeding support groups in promoting early initiation of breastfeeding within an hour of birth and EBF at 6 months in the six JP sites. In addition, we explored whether there was an interaction (or synergy) between peer counseling and membership in breastfeeding support groups to assess their joint effects on levels of early initiation of breastfeeding within an hour of birth and EBF at 6 months.

## Methods

### Research design and study population

The evaluation of the Joint Programme utilized a ‘before-and-after’ non-experimental type of study design. A baseline survey was carried out in early 2011 to establish the baseline levels of child undernutrition and prevalence of optimal breastfeeding practices [35]. Subsequently, the interventions were implemented in April 2011 to September 2012, shortly after the Baseline Survey. The Endline Survey was conducted in late 2012 to assess the effectiveness of JP interventions in improving the prevalence of optimal breastfeeding practices and in reducing undernutrition among the target children. We used data from the Endline Survey to examine the independent associations and possible interactions of two particular JP interventions (i.e., the peer counselors and the mother’s membership in breastfeeding support groups) with early initiation of breastfeeding and EBF among the target mothers.

There were six JP implementation sites in the Philippines: the cities of Naga, Iloilo, and Zamboanga, and the municipalities of Ragay in Camarines Sur; Carles in Iloilo; and Aurora in Zamboanga del Sur. A stratified two-stage systematic random sampling was employed to select the study participants. The *barangays* or villages in each city/municipality served as the primary sampling units (PSU), which were selected systematically with probability proportional to the PSU’s population size. Prior to sampling for the Endline Survey, 44 barangays in Zamboanga City were excluded because of security problems. From each remaining PSU, children less than 2 years old were randomly selected, with equal probability, using the lists of eligible children in each barangay. The lists of eligible children from each barangay were collected from local health workers and validated and updated for completeness by mappers/validators employed by the Endline Survey research team.

A sample size of 2584 mother-infant dyads from all six JP sites was required to detect a 3% absolute decrease, from the baseline, in the prevalence of underweight for age, using a 0.05 level of significance, 80% power, a design effect of 1.2, and an allowance of 10% for non-participation. However, only mothers who were the actual caregivers of the target children were included in our analyses. Data from other child caregivers, such as fathers, were excluded to minimize the effect of different qualities of recall that may arise from having different types of child caregivers. In the analysis for the outcome on EBF at 6 months, we only included infants aged at least 6 months since they were the only ones with data for the outcome of interest. Those younger than 6 months were excluded in the analysis for EBF as they were no longer followed for the outcome of interest.

### Operational definition of study variables and description of interventions of interest

The outcome variables in this study were early initiation of breastfeeding within 1 h of birth and EBF at 6 months. Infants were categorized based on the time of breastfeeding initiation: early initiation for those who were breastfed within 1h of birth and late initiation for those who were breastfed 1 h after birth. For EBF at 6 months, mothers were asked at what age, in months, solid foods or other liquids were first introduced to the infant. We dichotomized this variable into mothers who gave solid/liquid foods to the child 6 months after birth and mothers who did not.

The exposure variables in this study were home visit/s by a peer counselor during the mother’s prenatal period, home visit/s by a peer counselor after delivery, and membership of the mother in a breastfeeding support group during the index pregnancy. Being visited by a peer counselor during the prenatal period and membership of the mother in a breastfeeding support group during the index pregnancy were considered as exposure variables in the model for early initiation of breastfeeding. For EBF, we considered these two variables, together with home visit/s by a peer counselor after delivery as our exposure variables. All three are dichotomous variables (either visited by a peer counselor before and after delivery or not, and member of a support group or not). Details on the mobilization of peer counselors and breastfeeding support groups are presented in Additional file 1. The probable confounders in these associations of interest included place of residence, age of mother in years, total monthly household income, employment status of mother and partner, number of people living in household, number of living older siblings, mode of delivery of index child, birth attendant of index child, place of delivery, sex of child, maternal knowledge score, attendant during prenatal services, month of first prenatal service, and membership in the Pantawid Pamilyang Pilipino Program (4Ps). The 4Ps is a conditional cash transfer program implemented by the Philippine government which targets low-income Filipino families in return for complying with a set of conditions on children’s education and the family’s utilization of health services such as prenatal check-up and child vaccination [36]. In the model for EBF, early initiation of breastfeeding was also considered as a probable confounder.

### Data management and analysis

Data quality checks, such as checks for duplicates and range checks were performed on the dataset prior to any analysis. Quantitative variables, including age and monthly income, were recoded to allow the assessment of possible linear trends in the association between these variables and the outcome [37]. Some categorical variables were recoded to ensure that each stratum would have sufficient number of observations. Maternal knowledge score was aggregated from seven yes-no questions. Incorrect answers or “don’t know” answers were coded as incorrect and given a ‘0’ score, and ‘1’ for each correct answer. Scores ranged from 0 to 7, with higher scores implying better maternal knowledge. Lastly, variables that may be correlated were combined, such as marital status of the mother and employment status of partner. No observations were removed at any point in the analysis to ensure that standard errors can be computed correctly.

The exposure variables were cross-tabulated with each of the two outcome variables and the association of each of these exposures with each outcome variable were tested with Pearson’s χ2 test for categorical exposures, adjusted Wald test for normally distributed continuous variables, or Wilcoxon rank-sum test for skewed continuous variables. The distribution of missing data was shown for each variable but were not included in estimating the *p*-values. For each of these associations, crude odds ratios (cORs) were estimated using simple logistic regression for survey data. As part of screening potential confounders, each probable confounder and the outcome variable were cross-tabulated with each of the exposure variables using similar statistical tests as described above. Prior to doing multivariable analyses, observations with missing data for any of the variables of interest were excluded from the analysis. We used logistic regression methods for both early initiation of breastfeeding and EBF. Details of building the final models are presented in Additional file 2.

A level of significance of 0.05 was used in all analyses, and 95% Confidence Intervals (CIs) were reported. Data management and analyses were carried out in Stata 14.2 [38].

## Results

A total of 2542 parent-infant dyads were interviewed in the Endline Survey, giving a 98.4% response. Of these, only 2343 (93.1%) of the households had mothers who served as infant caregiver and were thus included in the analyses. Majority (77.6%) of the mothers were from urban areas. The mothers were between 15 and 50 years old, and most were married or living with their partners (92.2%). Most (92.3%) mothers were employed. During their index pregnancy, some of them (23.5%) were visited by a peer counselor and/or were reported as members of a breastfeeding support group (33.4%). When they gave birth to the index child, most of them (67.0%) delivered in health facilities and were attended by skilled professional birth attendants (75.4%). Some of them (28.5%) were also visited by peer counselors after delivery. Lastly, only 63.1% of mothers have initiated breastfeeding within an hour of birth. Out of the 2343 households who had mothers who served as the infant caregiver, 1865 (79.9%) were aged 6 months old or older. Out of this, only 34.7% had exclusively breastfed their children for 6 months (Table 1).

**Table 1:** Characteristics of the study participants (*n* = 2343)

Variable/Category	Frequency (%)
Visit by a peer counselor during prenatal period^a^	
No	1817 (75.5)
Yes	502 (23.5)
Visit by a peer counselor after delivery^a^	
No	1797 (71.1)
Yes	532 (28.5)
Membership in breastfeeding support groups^a^	
No	1683 (66.3)
Yes	650 (33.4)
Place of residence	
Urban area	2091 (77.6)
Rural area	252 (22.4)
Age of mother in years^a^	
15–19	179 (7.2)
20–24	634 (26.8)
25–29	634 (25.3)
30–34	521 (23.9)
35–39	246 (10.9)
40–50	120 (5.6)
Monthly income (PhP)	
0–3800	469 (21.3)
3801 – 5999	411 (16.6)
6000 – 8999	524 (21.0)
9000 – 15,999	480 (20.8)
16,000+	459 (20.4)
Employment status of mother	
Employed	2167 (92.3)
Unemployed	176 (7.7)
Employment status of partner^a^	
Employed	2096 (88.7)
Unemployed	85 (3.8)
Marital status^a^	
Married/Living together	2176 (92.2)
Never married/separated/divorced/widowed	163 (7.6)
Combined variable for marital status and employment status of partner^a^	
Single mother	163 (7.6)
Has employed partner/spouse	2090 (88.4)
Has unemployed partner/spouse	85 (3.8)
Membership in a conditional cash incentive program of the government (4Ps)^a^	
No	1853 (76.8)
Yes	487 (23.1)
Prenatal care provider^a^	
Doctor/Nurse/Midwife	2271 (96.3)
None/Traditional Birth Attendant	61 (3.5)
Mode of delivery^a^	
Normal	2139 (90.8)
Caesarean/other	195 (8.6)
Birth attendant^a^	
Skilled	1805 (75.4)
Traditional birth attendant/none/self/relatives/underboard midwife	512 (23.7)
Place of delivery^a^	
Home-based	667 (32.2)
Government healthcare facility	1434 (57.4)
Private healthcare facility	225 (9.6)
Sex of child	
Boy	1182 (51.5)
Girl	1161 (48.5)
Initiation of breastfeeding within one hour of birth^a^	
Early	1500 (63.1)
Late	763 (35.2)
Exclusive breastfeeding at six months^a,b^	
No	1155 (62.7)
Yes	621 (34.7)

^a^Has missing data

^b^*n* = 1865 infants who are at least six months old

The distribution of the mothers’ age of gestation at their first prenatal checkup was right-skewed as most mothers had their first visit during the first 3 months of pregnancy (median = 3). The knowledge scores of the respondents ranged from 0 to 7 and these scores were left-skewed in distribution as most mothers had scores of 5 or higher (median = 6). The number of living older siblings of the infants in the study ranged from 0 to 13. The distribution of this variable was right-skewed as the infants generally had few older siblings (median = 1). The household size of the respondents ranged from 2 to 25 members. This variable was also right-skewed, as the respondents lived in relatively smaller households (median = 6).

### Associations with early initiation of breastfeeding

Among the probable confounders considered, place of residence, maternal age, monthly income, membership in 4Ps, mode of delivery, birth attendant, and place of delivery were all found to have strong evidence of association with early initiation of breastfeeding. Maternal knowledge score and number of older siblings alive were also found to be significantly associated with early initiation of breastfeeding (Table 2). Without adjusting for confounding, visits by peer counselors during the prenatal period (cOR 1.48; 95% CI 1.16, 1.88) and membership in breastfeeding support groups (cOR 1.56; 95% CI 1.17, 2.08) were significantly associated with early initiation of breastfeeding (Table 3).

**Table 2:** Cross-tabulations of exposure and probable confounding variables with early initiation of breastfeeding within one hour of birth (*n* = 2343)

Variable/Category	Initiation of breastfeeding^**a**^		***p***-value
Visit by a peer counselor during prenatal period	Late	Early	< 0.01
No	611 (37.3)	1138 (60.8)	
Yes	147 (29.1)	346 (70.1)	
Membership in breastfeeding support groups			< 0.01
No	580 (38.4)	1032 (59.6)	
Yes	181 (29.0)	463 (70.1)	
Place of residence			0.03
Urban area	707 (38.1)	1304 (59.8)	
Rural area	56 (25.3)	196 (74.8)	
Age of mothers in years			< 0.01
15–19	52 (32.3)	119 (65.2)	
20–24	214 (37.0)	402 (61.5)	
25–29	193 (33.3)	426 (65.9)	
30–34	197 (41.8)	303 (56.2)	
35–39	65 (24.2)	170 (72.8)	
40–50	40 (32.9)	75 (65.8)	
Monthly income (PhP)			< 0.01
0–3800	129 (30.6)	322 (67.4)	
3801 – 5999	110 (27.1)	287 (72.0)	
6000 – 8999	185 (39.4)	322 (58.8)	
9000 – 15,999	152 (34.4)	311 (64.1)	
16,000+	187 (43.2)	258 (55.0)	
Employment status of mother			0.97
Employed	701 (35.2)	1392 (63.1)	
Unemployed	62 (35.3)	108 (63.6)	
Employment status of partner			0.46
Employed	681 (35.6)	1343 (62.8)	
Unemployed	33 (40.4)	50 (58.7)	
Marital status			0.20
Married/Living together	712 (35.7)	1390 (62.6)	
Never married/separated/divorced/widowed	50 (29.7)	107 (68.5)	
Combined variable for civil status and employment status of partner			0.32
Single mother	50 (29.7)	107 (68.5)	
Has employed partner/spouse	679 (35.6)	1339 (62.8)	
Has unemployed/partner/spouse	33 (40.4)	50 (58.7)	
Membership in 4Ps			0.04
No	614 (36.7)	1171 (61.5)	
Yes	148 (30.2)	327 (68.9)	
Prenatal care provider			0.75
Doctor/Nurse/Midwife	741 (35.4)	1457 (63.1)	
None/Traditional Birth Attendant	20 (32.3)	37 (63.7)	
Mode of delivery			< 0.01
Normal	627 (32.0)	1446 (66.7)	
Caesarean/other	132 (68.9)	49 (26.8)	
Birth attendant			0.02
Skilled	606 (37.1)	1137 (61.0)	
Traditional birth attendant/none/self/relatives/underboard midwife	149 (29.1)	348 (69.9)	
Place of delivery			< 0.01
Home-based	192 (28.5)	456 (70.6)	
Government healthcare facility	462 (36.7)	926 (61.8)	
Private healthcare facility	103 (47.9)	110 (57.9)	
Sex of child			0.72
Boy	381 (34.7)	763 (63.6)	
Girl	382 (35.8)	737 (62.7)	
Month when prenatal care was first availed (*n* = 2274)			0.70^b^
Maternal knowledge score (*n* = 2202)			< 0.01^b^
Number of living older siblings (*n* = 2338)			0.01^b^
Household size (*n* = 2343)			0.99^b^

^a^Has missing data so frequencies are not equal to 2343 and row percentages are not equal to 100%

^b^*p*-value from Wilcoxon rank-sum test

**Table 3:** Crude and adjusted association of peer counselor visits during prenatal period and membership in breastfeeding support groups with early initiation of breastfeeding within 1 h of birth

Variable/Category	cOR and 95%	***p***-value	aOR and 95% CI^**a**^	***p***-value
Visit by a peer counselor during prenatal period				
No	1 (baseline)		1 (baseline)	
Yes	1.48 (1.16, 1.88)	< 0.01	1.32 (0.96, 1.80)	0.08
Membership in breastfeeding support groups				
No	1 (baseline)		1 (baseline)	
Yes	1.56 (1.17, 2.08)	< 0.01	1.49 (1.12, 1.98)	< 0.01
Age group				
15–19	1 (baseline)		1 (baseline)	
20–24	0.82 (0.58, 1.16)	0.26	0.85 (0.55, 1.30)	0.44
25–29	0.98 (0.68, 1.40)	0.91	1.08 (0.66, 1.77)	0.74
30–34	0.67 (0.46, 0.95)	0.03	0.71 (0.41, 1.24)	0.23
35–39	1.49 (0.96, 2.32)	0.08	1.23 (0.68, 2.24)	0.48
40–50	0.99 (0.58, 1.69)	0.97	0.81 (0.39, 1.68)	0.22
Monthly income				
0–3800	1 (baseline)		1 (baseline)	
3801 – 5999	1.21 (0.76, 1.92)	0.42	1.41 (0.89, 2.23)	0.14
6000 – 8999	0.68 (0.47, 0.97)	0.04	0.83 (0.60, 1.15)	0.26
9000 – 15,999	0.85 (0.59, 1.21)	0.36	1.16 (0.78, 1.72)	0.46
16,000+	0.58 (0.40, 0.84)	< 0.01	0.83 (0.61, 1.13)	0.22
Combined variable for place of delivery and birth attendant				
Home birth; skilled birth attendant	1 (baseline)		1 (baseline)	
Home birth; unskilled birth attendant	1.05 (0.64, 1.71)	0.85	0.73 (0.40, 1.34)	0.31
Government healthcare facility birth; skilled birth attendant	0.71 (0.45, 1.12)	0.14	0.84 (0.51, 1.38)	0.49
Private healthcare facility birth; skilled birth attendant	0.41 (0.23, 0.72)	< 0.01	0.61 (0.30, 1.24)	0.17
Number of living older siblings of the index child	1.07^b^ (1.01, 1.15)	0.03	1.04 (0.95, 1.13)	0.40
Maternal knowledge score	1.33^b^ (1.19, 1.48)	< 0.01	1.30 (1.13, 1.49)	< 0.01
Mode of delivery				
Normal	1 (baseline)		1 (baseline)	
Caesarean/other	0.19 (0.12, 0.28)	< 0.01	0.20 (0.13, 0.32)	< 0.01
Place of residence				
Urban	1 (baseline)		1 (baseline)	
Rural	1.89 (1.06, 3.35)	0.03	1.54 (0.90, 2.62)	0.11
Employment status of mother				
Employment	1 (baseline)		1 (baseline)	
Unemployment	1.01 (0.70, 1.44)	0.97	0.97 (0.68,1.38)	0.86
Sex of child				
Boy	1 (baseline)		1 (baseline)	
Girl	0.96 (0.75, 1.22)	0.71	1.07 (0.82, 1.39)	0.63

^a^Adjusted for other variables in the table

^b^Common odds ratio showing increase in odds per unit increase in level of the variable

Among the probable confounders, place of residence, maternal age, household size, number of older siblings alive, monthly income, mother’s employment status, marital status, the combined variable of marital status and partner employment status, membership in 4Ps, birth attendant, and place of delivery were all associated with peer counselor visits during the prenatal period (Additional file 3). Meanwhile, household size, number of older siblings alive, maternal age, and membership in 4Ps were all associated with membership in a breastfeeding support group (Additional file 4). Therefore, place of residence, maternal age, monthly income, membership in 4Ps, birth attendant, place of delivery, and number of living older siblings may confound the association between the exposure variables and early initiation of breastfeeding. However, we forced other variables (e.g., maternal knowledge, mode of delivery, mother’s employment status, and sex of child) in our regression model for early initiation of breastfeeding because these variables were found to be ‘a-priori’ determinants of this outcome in the literature.

In building our final regression model, we used the combined variable for civil status and partner’s employment status to minimize missing data. We also detected collinearity between place of delivery and birth attendant, so we combined place of delivery and birth attendant, and used this variable in building our final regression model. To ensure that models were comparable during model-building, we excluded from the multivariable analyses some 316 observations with missing data in any of the remaining variables of interest. Thus, only 2027 (87.5%) respondents were included in the final analysis.

In our final regression model for early initiation of breastfeeding, there was a departure from the linearity assumption for maternal age (*p* = 0.02) and monthly income (*p* < 0.01). Stratum-specific adjusted odds ratios (aORs) for these variables are presented in the tables. However, there was no evidence of interaction between prenatal visits by a peer counselor and membership in breastfeeding in support groups on early initiation of breastfeeding (*p* = 0.20), thus we did not include interaction terms in our final models. After adjusting for confounders, the odds of early initiation of breastfeeding is 1.32 (95% CI 0.96, 1.80) times higher among mothers who were visited by a peer counselor during their prenatal period compared to those who were not visited. On the other hand, members of breastfeeding support groups have 49% greater odds (OR 1.49; 95% CI 1.12, 1.98) of initiating breastfeeding within 1 h of child’s birth compared to non-members of breastfeeding support groups. Thus, there is insufficient evidence to show that visits by a peer counselor was associated with increased odds of early initiation of breastfeeding, but there is strong evidence that membership in breastfeeding support groups was associated with early initiation of breastfeeding.

### Associations with EBF at six months

Among the probable confounders considered, only birth attendant, and early initiation of breastfeeding were found to have strong evidence of association with EBF, while household size and prenatal care provider were of borderline significance (Table 4). Without adjusting for confounding, visits by peer counselors during pregnancy increased the odds of EBF by 15% (cOR 1.15; 95% CI 0.90, 1.47), and visits by peer counselors after delivery increased the odds of EBF by 28% (cOR 1.28; 95% CI 0.97, 1.70), but the evidence for these associations were not strong. Meanwhile, membership in breastfeeding support groups (cOR 1.73; 95% CI 1.32, 2.27) was strongly associated with EBF (Table 5).

**Table 4:** Cross-tabulations of exposure and probable confounding variables with exclusive breastfeeding at 6 months

Variable/Category	Exclusive breastfeeding at six months^a^		***p***-value
Visit by a peer counselor during prenatal period	No	Yes	0.27
No	895 (63.2)	469 (34.0)	
Yes	248 (60.9)	148 (37.6)	
Membership in breastfeeding support groups			< 0.01
No	858 (66.7)	410 (30.5)	
Yes	292 (54.8)	210 (43.3)	
Visited by a peer counselor after delivery			0.09
No	890 (64.0)	456 (33.0)	
Yes	258 (59.4)	163 (39.2)	
Place of residence			0.18
Urban area	1040 (63.9)	531 (32.9)	
Rural area	115 (58.8)	90 (40.7)	
Age of mothers in years			0.81
15–19	81 (65.6)	38 (29.5)	
20–24	308 (62.9)	170 (34.3)	
25–29	315 (62.7)	168 (36.3)	
30–34	256 (60.5)	143 (35.9)	
35–39	125 (61.8)	73 (36.1)	
40–50	65 (68.6)	28 (29.8)	
Monthly income (PhP)			0.20
0–3800	223 (59.7)	133 (37.8)	
3801 – 5999	186 (59.2)	125 (38.3)	
6000 – 8999	266 (61.8)	149 (36.6)	
9000 – 15,999	242 (66.2)	117 (31.3)	
16,000+	238 (66.5)	97 (29.9)	
Employment status of mother			0.98
Employed	1065 (62.7)	578 (34.7)	
Unemployed	90 (63.1)	43 (34.8)	
Employment status of partner			0.56
Employed	1025 (62.5)	557 (35.0)	
Unemployed	43 (59.0)	25 (39.3)	
Marital status			0.18
Married/Living together	1065 (62.3)	581 (35.2)	
Never married/separated/divorced/widowed	87 (68.1)	39 (29.2)	
Combined variable for civil status and employment status of partner			0.37
Single mother	87 (68.1)	39 (29.2)	
Has employed partner/spouse	1021 (62.4)	556 (35.0)	
Has unemployed partner/spouse	43 (59.0)	25 (39.3)	
Membership in 4Ps			0.41
No	922 (63.4)	469 (34.1)	
Yes	232 (60.8)	151 (36.8)	
Prenatal care provider			0.05
Doctor/Nurse/Midwife	1117 (62.2)	611 (35.3)	
None/Traditional Birth Attendant	33 (76.8)	9 (19.9)	
Mode of delivery			0.66
Normal	1050 (62.7)	575 (35.1)	
Caesarean/other	99 (62.0)	43 (31.4)	
Birth attendant			0.01
Skilled	897 (64.6)	468 (32.6)	
Traditional birth attendant/none/self/relatives/underboard midwife	247 (56.9)	147 (41.5)	
Place of delivery			0.45
Home-based	325 (60.5)	187 (37.5)	
Government healthcare facility	702 (64.1)	385 (33.7)	
Private healthcare facility	120 (63.6)	45 (30.6)	
Sex of child			0.35
Boy	595 (64.1)	307 (33.5)	
Girl	560 (61.3)	314 (36.1)	
Early initiation of breastfeeding			< 0.01
Late	436 (72.2)	153 (26.6)	
Early	718 (59.2)	468 (39.9)	
Month when prenatal care was first availed			0.14^b^
Maternal knowledge score			0.19^b^
Number of living older siblings			0.27^b^
Household size			0.05^b^

^a^Has missing data so frequencies are not equal to 2343 and row percentages are not equal to 100%

^b^*p*-value from Wilcoxon rank-sum test

**Table 5:** Crude and adjusted association of peer counselor visits during prenatal period, membership in breastfeeding support groups, and peer counselor visits after delivery with exclusive breastfeeding at six months

Variable/Category	cOR and 95% CI	***p***-value	aOR and 95% CI	***p***-value
Visit by a peer counselor during pre-natal period				
No	1 (baseline)		1 (baseline)	
Yes	1.15 (0.90, 1.47)	0.27	0.88 (0.65, 1.20)	0.42
Membership in breastfeeding support groups				
No	1 (baseline)		1 (baseline)	
Yes	1.73 (1.32, 2.27)	< 0.01	1.65 (1.20, 2.24)	< 0.01
Visit by a peer counselor after delivery				
No	1 (baseline)		1 (baseline)	
Yes	1.28 (0.97, 1.70)	0.09	1.14 (0.80, 1.63)	0.47
Initiation of breastfeeding				
Late	1 (baseline)		1 (baseline)	
Early	1.83 (1.32, 2.53)	< 0.01	2.07 (1.48, 2.89)	< 0.01
Combined variable for place of delivery and birth attendant				
Home birth; skilled birth attendant	1 (baseline)		1 (baseline)	
Home birth; unskilled birth attendant	1.74 (1.07, 2.82)	0.03	1.46 (0.87, 2.43)	0.15
Government healthcare facility birth; skilled birth attendant	1.27 (0.78, 2.06)	0.33	1.16 (0.70, 1.91)	0.56
Private healthcare facility birth; skilled birth attendant	1.17 (0.65, 2.09)	0.59	1.18 (0.62, 2.25)	0.62
Household size	0.94^b^ (0.89, 0.99)	0.03	0.92^b^ (0.85, 0.99)	0.03
Prenatal care provider				
Doctor/Nurse/Midwife	1 (baseline)		1 (baseline)	
None/Traditional Birth Attendant	1.16 (0.90, 1.49)	0.25	1.08 (0.16, 7.55)	0.94
Number of living older siblings	1.02^b^ (0.95, 1.09)	0.59	1.04^b^ (0.94, 1.15)	0.45
Mode of delivery				
Normal	1 (baseline)		1 (baseline)	
Caesarean/other	0.91 (0.58, 1.42)	0.67	1.27 (0.79, 2.06)	0.32
Age group	1.00^b^ (0.98, 1.02)		0.99^b^ (0.96, 1.02)	0.47
Sex of child				
Boy	1 (baseline)		1 (baseline)	
Girl	1.13 (0.87, 1.45)	0.35	1.20 (0.94, 1.55)	0.15
Combined variable for civil status and employment status of partner				
Single mother	1 (baseline)		1 (baseline)	
Has employed partner/spouse	1.31 (0.87, 1.98)	0.20	1.17 (0.62, 2.23)	0.63
Has unemployed partner/spouse	1.56 (0.79, 3.08)	0.20	1.62 (0.76, 3.45)	0.21
Monthly income	1.00 (1.00, 1.00)	0.04	1.00 (1.00, 1.00)	0.76
Employment status of mother				
Employed	1 (baseline)		1 (baseline)	
Unemployed	0.99 (0.65, 1.53)	0.98	1.07 (0.52, 2.19)	0.86

^a^Adjusted for other variables in the table

^b^Common odds ratio showing increase in odds per unit increase in level of the variable

Aside from the probable confounders which were identified to be associated with peer counselor visits during pregnancy, we also found the variables place of residence, prenatal care provider, and early initiation of breastfeeding to be strongly associated with peer counselor visits after delivery (Additional file 5). Thus, birth attendant, household size, early initiation of breastfeeding, and prenatal care provider satisfied the definition of a confounder in our data and would be included in the EBF model. Other known confounders from the literature, such as maternal civil status, mode of delivery, maternal age, number of living older siblings, and monthly income, and employment status of mother, and sex of infant, were also forced into the model. As with the early initiation of breastfeeding model, we used the combined variable of maternal marital status and partner employment, and the combined variable of place of delivery and birth attendant in modelling the association between receiving JP interventions (prenatal and postnatal counselor visits, and breastfeeding support groups) and exclusive breastfeeding. We also excluded from the regression analysis some 276 respondents with incomplete data in all the remaining variables of interest, thus our final sample for this analysis was 1589 (86.7%).

In our final model for EBF, we noted that there was no departure from the linearity assumption for age group (*p* = 0.88) and monthly income (*p* = 0.93); thus, only common aORs were reported. There were also no significant two-way or three-way statistical interactions between prenatal peer counselor visits, postnatal peer counselor visits, and membership in breastfeeding support groups which could affect EBF. Specifically, there were also no significant statistical interactions (or synergy) between prenatal and postnatal peer counselor visits (*p* = 0.35), as well as between prenatal peer counselor visits and membership in breastfeeding support groups (*p* = 0.16), postnatal peer counselor visits and membership in breastfeeding support groups (*p* = 0.74), and the three interventions with each other (*p* = 0.56) on exclusive breastfeeding. After adjusting for confounders, we report that there was no strong evidence that visits by peer counselors during pregnancy (aOR 0.88; 95% CI 0.65, 1.20) as well as visits after delivery (aOR 1.14; 95% CI 0.80, 1.63) were associated with EBF. However, being a member in breastfeeding support groups significantly increased the odds of EBF by 65% (aOR 1.65; 95% CI 1.20, 2.24) (Table 5).

## Discussion

Our secondary analysis of the Endline Survey data of the JP reveals that 63% of mothers breastfed within an hour of birth, and only 35% exclusively breastfed at 6 months, which are both below the DOH targets of 90% prevalence for early initiation of breastfeeding and 70% prevalence for EBF. While there is no strong evidence that visits by a peer counselor during pregnancy and after delivery are associated with early initiation of breastfeeding and EBF, there is strong evidence that membership in breastfeeding support groups is positively associated with both early initiation of breastfeeding and EBF. One possible explanation of why membership in breastfeeding support groups was effective in promoting both early initiation of breastfeeding and EBF, while peer counselors only had marginal effectiveness, may be the active knowledge transfer during breastfeeding support group sessions compared to the relatively passive one-on-one lectures in peer counseling sessions. Our secondary analysis did not detect possible synergy (i.e., statistical interaction) between peer counselor visits and membership in breastfeeding support groups, and early initiation of breastfeeding and EBF at 6 months. In other words, the effect of these two specific JP interventions on early initiation of breastfeeding and EBF are probably independent of each other.

Home visits by a peer counselor are designed to encourage mothers to get adequate prenatal care and to advocate for them, and promote the positive effects of early initiation of breastfeeding and EBF. The mothers were also coached by these peer counselors to continue breastfeeding exclusively up to 6 months after birth, to introduce quality complementary food only after 6 months, and to breastfeed beyond 2 years. Breastfeeding support groups, on the other hand, were organized in the communities to promote proper IYCF practices among the mothers with infants and children less than 2 years old. These support groups were supposed to encourage and support the mothers so they can initiate breastfeeding early and continue breastfeeding their infants exclusively until the baby is 6 months old. Together with proper complementary feeding, members of the support groups motivate each other to continue breastfeeding the babies up to 2 years and beyond, if possible, to maximize the positive effects of mothers’ milk on young children.

The importance of family- or community-based interventions to improve neonatal and child health cannot be overemphasized. There is evidence that community-based interventions can reduce all-cause neonatal mortality by 10–50% [39]. However, studies assessing the effectiveness of these interventions are few and the findings are contradictory [40, 41]. There are also reviews on community-based interventions, optimal feeding practices, and child health. A Cochrane review reported that there is no strong evidence to conclude that non-healthcare professional-led interventions, which include support groups and peer counselors, affect early initiation of breastfeeding among target mothers [42]. Another review identified the following gaps in operations research for community-based interventions to promote child health: [a] how health workers, including non-healthcare professionals, could most effectively deliver the needed services for newborns and children at the community; [b] the scope of service of community health workers; [c] ways to link community health workers with referral facilities to provide care for mothers and children; and [d] how community-based interventions can be managed sustainably [43]. A third review reported that interventions conducted by lay people, especially those providing emotional support and counseling, including services that are given during pregnancy and continued postpartum, are associated with greater odds of EBF. The review concluded that effective interventions to promote EBF should have multiple components, have a clear training protocol to engage both professionals and non-professionals healthcare providers, and have continuity of service between the health facility and the community [44]. The fact that these studies have different results and recommendations could be probably explained by differences in the interventions, the target populations, and the study methods employed.

Our study has a number of important limitations. First, the exact nature of the engagement of peer counselors with the target mothers was not clearly defined and this may explain the lack of association seen between peer counselor visits and early initiation of breastfeeding and EBF. Notably, a fifth of the peer counselors mentioned that they have little or no knowledge on their roles and activities, while another one-third felt that they have inadequate knowledge on what to do if a child is sick [15]. Second, while breastfeeding support groups enjoyed greater acceptance than one-on-one visits by peer counselors, there were also some issues including implementation delays, issues in training and engagement, and the non-mandatory participation of the members. Third, the sample size used in the analyses may have been insufficient for tests of statistical interactions [45]. This partly explains the absence of a statistically significant effect of the hypothesized synergy between the JP interventions that we studied on both early initiation of breastfeeding or EBF at 6 months. Fourth, in assessing the effectiveness of community interventions, a cluster randomized trial would be the more appropriate design to use [46]. However, since the main objective of the JP was to decrease the prevalence of undernutrition and improve the prevalence of optimal breastfeeding practices, and not to assess the effectiveness of interventions, a before-and-after evaluation design with two separate cross-sectional studies was used instead given the available resources. As a result, the issue of reverse causality, which is inherent in cross-sectional studies, may affect the internal validity of our analysis [47]. Fifth, differences in the extent of missing outcome data for our different exposure variables could point to possible selection bias: [a] percent of missing early initiation of breastfeeding data is higher among those who were not visited by a peer counselor during prenatal period compared to those who were visited; [b] percent of missing EBF data is higher among members of breastfeeding support groups than those who were not; and [c] percent of those with missing EBF data is higher among those who were visited by a peer counselor after delivery compared to those who were not visited. Sixth, our study may also suffer from information bias since we utilized self-reported data. The findings of our study are, therefore, only as good as the reports of the mothers who took part in the study. Mothers of relatively older infants may be less likely to provide accurate recall as compared to mothers of younger infants and/or newborns [48]. Our study may also have residual confounding because important correlates of the outcome such as educational status of the mother and opinion of other family members were not collected [28]. Lastly, the Endline Survey did not have data on the number of visits of peer counselors, as well as the number of sessions held by the breastfeeding support groups, which prevented us from characterizing the nature of mother-peer counselor dynamics. This information gap also prevented us from exploring possible dose-response relationships between the JP interventions and the outcome variables. Despite this, we controlled for the effect of other important confounders like mode of delivery, maternal age, number of living older siblings, place of delivery and birth attendant.

## Conclusions

Our findings suggest that early initiation of breastfeeding within 1 hour of birth and EBF at 6 months in the country do not meet national targets. We also found that there is insufficient evidence that peer counseling during pregnancy and after delivery are associated with early initiation of breastfeeding and EBF, but that there is strong evidence that membership in breastfeeding support groups is positively associated with both early initiation of breastfeeding and EBF at 6 months. Adopting good training designs for community volunteers and conducting regular sessions may make breastfeeding support groups more effective in improving early initiation of breastfeeding and EBF practices. While the role of peer counselors in promoting early initiation of breastfeeding and EBF remains unclear, they may still have a significant role to play in promoting both. We recommend that peer counselors should have clear messages to deliver to target mothers to promote early initiation of breastfeeding, exclusive breastfeeding, and to discuss the importance of prenatal care [49]. Although trained healthcare professionals like midwives and nurses were reported to be more effective in promoting breastfeeding among target mothers, there are not enough of them to do this [42, 44]. This is one area where lay community health workers like breastfeeding counselors can be tapped to carry out some of these functions. Setting performance targets coupled with close supervision, salaries, or incentives are necessary to institutionalize grassroots-level interventions to promote any community-based intervention including optimal breastfeeding practices. These interventions should be supported by the government at all levels through financing, partnerships between local and external stakeholders, and logistical support at all levels to further ensure effective and efficient implementation [50].

Our suggestion to integrate non-healthcare professionals in efforts to promote early initiation of breastfeeding and EBF could be tested further in future intervention studies. Operations research can address various information gaps on child health which could be addressed by doing community trials [51]. This has been emphasized by one Cochrane review which concluded that current evidence on the effectiveness of non-healthcare professional-led interventions on early initiation of breastfeeding are few and of poor quality [42]. Thus, methodologically sound studies to assess the effectiveness of peer counselors and/or support groups in promoting optimal breastfeeding behaviors are still needed. The need for more research on this topic to influence policies and programs is demonstrated by the low prevalence of early initiation of breastfeeding and exclusive breastfeeding worldwide, including the Philippines.

## Supplementary Information

**Additional file 1.** Detailed description of assessed interventions. A more detailed description of the interventions (peer counselor visits before and after delivery and breastfeeding support groups) being assessed containing recruitment procedures, training and compensation, and other implementation details.

**Additional file 2.** Regression analyses. Detailed description of how regression analyses was carried out, including variable selection procedures.

**Additional file 3.** Cross-tabulations and crude odds ratios of categorical variables with visit by a peer counselor during prenatal period. Cross-tabulations and crude odds ratios of outcomes and probable confounders with visit by peer counselor during prenatal period as part of the assessment of potential confounding effects.

**Additional file 4.** Cross-tabulations and crude odds ratios of categorical variables with membership in breastfeeding support groups. Cross-tabulations and crude odds ratios of outcomes and probable confounders with membership in breastfeeding support groups as part of the assessment of potential confounding effects.

**Additional file 5.** Cross-tabulations and crude odds ratios of categorical variables with visit by a peer counselor after delivery. Cross-tabulations and crude odds ratios of outcomes and probable confounders with visit by a peer counselor after delivery as part of the assessment of potential confounding effects.

## Data Availability

The datasets used and/or analyzed during the current study are available from the corresponding author (opsaniel@up.edu.ph) on reasonable request.

## Abbreviations

4Ps: Pantawid Pamilyang Pilipino Program
aOR: Adjusted odds ratio
cOR: Crude odds ratio
DOH: Department of Health
EBF: Exclusive breastfeeding at six months of age
FAO: Food and Agriculture Organization
ILO: International Labor Organization
IYCF: Infant and young child feeding
MDG-FJP: Millennium Development Goals Achievement Fund – Joint Programme on Ensuring Food Security and Nutrition for Children 0–24 months old in the Philippines
MDG: Millennium Development Goal
OR: Odds ratio
UNICEF: United Nations Children’s Fund
WFP: World Food Program
WHO: World Health Organization
