# Supplementary material for: Effectiveness of peer counseling and membership in breastfeeding support groups in promoting optimal breastfeeding behaviors in the Philippines
Source: Int Breastfeed J. 2021 Jul 12;16:53. doi: 10.1186/s13006-021-00400-5 (PMC8274007; doi:10.1186/s13006-021-00400-5)
Supplement: Supplementary file 1 — Additional file 1. Detailed description of assessed interventions. A more detailed description of the interventions (peer counselor visits before and after delivery and breastfeeding support groups) being assessed containing recruitment procedures, training and compensation, and other implementation details. [file 13006_2021_400_MOESM1_ESM.docx]

**Additional File 1: Detailed description of assessed interventions**

Mobilization of peer counselors and breastfeeding support groups were among the interventions implemented by the JP. Peer counselors were volunteers, often female local community workers, who were engaged and trained to educate mothers about EBF, correct positioning and attachment of the baby during breastfeeding, visit pregnant and post-partum mothers at home to advocate breastfeeding, orient pregnant mothers about the Milk Code which prohibits advertisements of breastmilk substitutes, and teach pregnant mothers how to prepare for their delivery. These peer counselors were trained for five days by the Programme staff (three days to administer the interview schedule and two days of supervised mock interviews in the field) and asked to cover several households in a barangay (i.e., village) or part of a large barangay. They were expected to obtain contact details of pregnant mothers or mothers who have recently delivered. Meanwhile, breastfeeding support groups were led by female influential community members who engaged target mothers in small group discussions about many topics related to breastfeeding including the benefits of breastmilk, advantages of breastfeeding, correct breastfeeding techniques, proper diet of lactating mothers, and proper complementary feeding. Like the peer counselors, the leaders of breastfeeding support groups were likewise trained by local Programme implementors.
