# Supplementary material for: Effectiveness of peer counseling and membership in breastfeeding support groups in promoting optimal breastfeeding behaviors in the Philippines
Source: Int Breastfeed J. 2021 Jul 12;16:53. doi: 10.1186/s13006-021-00400-5 (PMC8274007; doi:10.1186/s13006-021-00400-5)
Supplement: Supplementary file 2 — Additional file 2. Regression analyses. Detailed description of how regression analyses was carried out, including variable selection procedures. [file 13006_2021_400_MOESM2_ESM.docx]

**Additional File 2: Regression Analyses**

We ran two logistic regression models, one for each outcome variable. In building the final models, the main exposure variables were fitted first. Afterwards, important confounders were fitted into the model. After this, any variable deemed to be an important confounder based on the literature (even if they have not shown any strong association with the exposure or outcome in this dataset) was forced into the model. Any remaining variables were fitted into the model one by one, starting with the smallest p-value in their respective cross-tabulations with the outcome. If any of these variables changed the estimate of the OR for any of the main exposure variables by >10%, then they were retained in the final model; otherwise, they were excluded.

Once grouped quantitative variables were fitted into the model, test for departure from the linearity assumption was carried out by observing the stratum specific odds ratios (OR), and by doing an adjusted Wald test. If there is departure from the linearity assumption, stratum specific ORs were presented. Otherwise, a common estimate for the linear effect of the exposure variable on the outcome was reported (36). After testing for departure from the linearity assumption of grouped quantitative variables, interaction between the exposure variables were assessed (37). Any significant interaction parameters were shown, and linear combinations were used to estimate interaction parameters.
