# Supplementary material for: Effectiveness of peer counseling and membership in breastfeeding support groups in promoting optimal breastfeeding behaviors in the Philippines
Source: Int Breastfeed J. 2021 Jul 12;16:53. doi: 10.1186/s13006-021-00400-5 (PMC8274007; doi:10.1186/s13006-021-00400-5)
Supplement: Supplementary file 3 — Additional file 3. Cross-tabulations and crude odds ratios of categorical variables with visit by a peer counselor during prenatal period. Cross-tabulations and crude odds ratios of outcomes and probable confounders with visit by peer counselor during prenatal period as part of the assessment of potential confounding effects. [file 13006_2021_400_MOESM3_ESM.docx]

Additional File 3. Cross-tabulations and crude odds ratios of categorical variables with visit by a peer counselor during prenatal period.

|  | Visit by peer counselor during prenatal period | | | p-value | Crude OR | p-value of crude OR |
| --- | --- | --- | --- | --- | --- | --- |
| Membership in  breastfeeding support groups | No | Yes | Missing | <0.01 |  |  |
| No | 1,443 (84.3) | 230 (15.1) | 10 (0.6) |  | 1  (baseline) |  |
| Yes | 374 (58.7) | 272 (40.5) | 4 (0.9) |  | 3.87  (2.74-5.46) | <0.01 |
| *Missing* | 0 (0.0) | 0 (0.0) | 10 (1.0) |  |  |  |
| Peer counselor visit after delivery |  |  |  | <0.01 |  |  |
| No | 1554 (85.2) | 235 (14.3) | 8 (0.5) |  | 1  (baseline) |  |
| Yes | 261 (52.1) | 267 (47.0) | 4 (0.9) |  | 5.37  (3.89-7.43) | <0.01 |
| *Missing* | 2 (12.6) | 0 (0.0) | 12 (87.5) |  |  |  |
| Place of residence |  |  |  | 0.01 |  |  |
| Urban area | 1,651 (77.7) | 418 (21.3) | 22 (1.1) |  | 1  (baseline) |  |
| Rural area | 166 (68.1) | 84 (31.2) | 2 (0.7) |  | 1.67  (1.11-2.50) | 0.01 |
| Age of mothers in years |  |  |  | 0.02 |  |  |
| 15-19 | 136 (71.5) | 43 (28.5) | 0 (0.0) |  | 1  (baseline) |  |
| 20-24 | 508 (79.7) | 120 (19.0) | 6 (1.3) |  | 0.60  (0.37-0.98) | 0.04 |
| 25-29 | 487 (74.7) | 144 (24.7) | 3 (0.6) |  | 0.83  (0.50-1.38) | 0.47 |
| 30-34 | 415 (77.2) | 102 (22.3) | 4 (0.5) |  | 0.73  (0.47-1.13) | 0.15 |
| 35-39 | 178 (66.9) | 67 (33.0) | 1 (0.01) |  | 1.24  (0.75-2.06) | 0.40 |
| 40-50 | 89 (74.4) | 26 (21.5) | 5 (4.1) |  | 0.73  (0.37-1.43) | 0.35 |
| *Missing* | 4 (70.3) | 0 (0.0) | 5 (29.8) |  |  |  |
| Monthly income (PhP) |  |  |  | 0.04 |  |  |
| 0 – 3,800 | 365 (79.3) | 97 (19.6) | 7 (1.1) |  | 1  (baseline) |  |
| 3,801 – 5,999 | 300 (66.9) | 108 (32.2) | 3 (0.9) |  | 1.95  (1.31-2.91) | <0.01 |
| 6,000 – 8,999 | 411 (73.7) | 108 (25.4) | 5 (1.0) |  | 1.39  (0.97-2.00) | 0.07 |
| 9,000 – 15,999 | 370 (76.9) | 106 (22.5) | 4 (0.6) |  | 1.18  (0.86-1.64) | 0.30 |
| 16,000+ | 371 (79.1) | 83 (19.6) | 5 (1.3) |  | 1.00  (0.64-1.57) | 0.98 |
| Employment status of mother |  |  |  | 0.04 |  |  |
| Employed | 1,676 (75.0) | 473 (24.3) | 18 (0.7) |  | 1  (baseline) |  |
| Unemployed | 141 (82.1) | 29 (14.3) | 6 (3.6) |  | 0.54  (0.30-0.96) | 0.04 |
| Employment status of partner |  |  |  | 0.09 |  |  |
| Employed | 1,616 (74.6) | 464 (24.7) | 16 (0.7) |  | 1  (baseline) |  |
| Unemployed | 71 (84.6) | 13 (13.4) | 1 (2.0) |  | 0.48  (0.20-1.17) | 0.10 |
| *Missing/Not applicable* | 130 (82.4) | 25 (14.4) | 7 (3.2) |  |  |  |
| Civil status |  |  |  | 0.04 |  |  |
| Married/Living together | 1,684 (75.1) | 475 (24.2) | 17 (0.8) |  | 1  (baseline) |  |
| Never married/separated/  divorced/widowed | 132 (82.9) | 24 (14.0) | 7 (3.1) |  | 0.53  (0.29-0.96) | 0.04 |
| *Missing* | 1 (2.7) | 3 (97.3) | 0 (0.0) |  |  |  |
| Combined variable for civil status and employment status of partner |  |  |  | 0.03 |  |  |
| Single mother | 132 (82.9) | 24 (14.0) | 7 (3.1) |  | 1  (baseline) |  |
| Has employed partner/  spouse | 1612 (74.6) | 462 (24.6) | 16 (0.7) |  | 1.95  (1.06-3.58) | 0.03 |
| Has unemployed  partner/spouse | 71 (84.6) | 13 (13.4) | 1 (2.0) |  | 0.94  (0.35-2.50) | 0.89 |
| *Missing* | 2 (26.4) | 3 (73.6) | 0 (0.0) |  |  |  |
| Membership in 4Ps |  |  |  | <0.01 |  |  |
| No | 1,469 (77.2) | 366 (21.9) | 18 (0.9) |  | 1  (baseline) |  |
| Yes | 345 (69.7) | 136 (28.9) | 6 (1.3) |  | 1.46  (1.19-1.91) | <0.01 |
| *Missing* | 3 (100.0) | 0 (0.0) | 0 (0.0) |  |  |  |
| Prenatal care provider |  |  |  | 0.43 |  |  |
| Doctor/Nurse/Midwife | 1,765 (75.5) | 492 (23.7) | 14 (0.7) |  | 1  (baseline) |  |
| None/Traditional Birth Attendant | 51 (80.6) | 10 (19.4) | 0 (0.0) |  | 0.77  (0.39-1.50) | 0.43 |
| *Missing* | 1 (5.9) | 0 (0.0) | 10 (94.1) |  |  |  |
| Mode of delivery |  |  |  | 0.62 |  |  |
| Normal | 1,655 (75.2) | 462 (23.8) | 22 (1.0) |  | 1  (baseline) |  |
| Caesarean/other | 154 (78.2) | 39 (21.6) | 2 (0.2) |  | 0.87  (0.51-1.51) | 0.62 |
| *Missing* | 8 (91.0) | 1 (9.1) | 0 (0.0) |  |  |  |
| Birth attendant |  |  |  | <0.01 |  |  |
| Skilled | 1,428 (77.6) | 367 (21.7) | 10 (0.7) |  | 1  (baseline) |  |
| Traditional birth  attendant/none/self/  relatives/underboard  midwife | 376 (69.4) | 132 (29.9) | 4 (0.7) |  | 1.54  (1.22-1.95) | <0.01 |
| *Missing* | 13 (59.7) | 3 (12.3) | 10 (28.0) |  |  |  |
| Place of delivery |  |  |  | <0.01 |  |  |
| Home-based | 496 (71.9) | 166 (27.3) | 5 (0.8) |  | 1  (baseline) |  |
| Government healthcare facility | 1,139 (78.9) | 286 (20.3) | 9 (0.8) |  | 0.68  (0.53-0.86) | <0.01 |
| Private healthcare facility | 176 (69.4) | 49 (30.6) | 0 (0.0) |  | 1.16  (0.75-1.81) | 0.50 |
| *Missing* | 1 (27.1) | 0 (0.0) | 10 (72.9) |  |  |  |
| Gender of child |  |  |  | 0.26 |  |  |
| Boy | 908 (74.2) | 260 (24.6) | 14 (1.2) |  | 1  (baseline) |  |
| Girl | 909 (76.9) | 242 (22.4) | 10 (0.7) |  | 0.88  (0.70-1.10) | 0.26 |
| Initiation of breastfeeding |  |  |  | <0.01 |  |  |
| Late | 611 (80.0) | 147 (19.4) | 5 (0.6) |  | 1  (baseline) |  |
| Early | 1,138 (72.7) | 346 (26.1) | 16 (1.2) |  | 1.48  (1.16-1.88) | <0.01 |
| *Missing* | 68 (87.4) | 9 (11.3) | 3 (1.2) |  |  |  |
| Exclusive breastfeeding |  |  |  | 0.23 |  |  |
| No | 895 (75.2) | 248 (23.8) | 12 (1.1) |  | 1  (baseline) |  |
| Yes | 469 (73.0) | 148 (26.5) | 4 (0.1) |  | 1.15  (0.90-1.47) | 0.27 |
| *Missing* | 74 (85.0) | 12 (14.0) | 3 (1.0) |  |  |  |
| Months when prenatal care was first availed | | | | 0.50^a^ | 0.91  (0.81-1.03) | 0.12 |
| Maternal knowledge score | | | | 0.11^a^ | 1.13  (0.98-1.30) | 0.08 |
| Household size | | | | <0.01^a^ | 1.02  (0.98-1.07) | 0.23 |
| Number of living older siblings | | | | 0.03^a^ | 1.05  (1.00-1.10) | 0.08 |

^a^ p-value from Wilcoxon rank-sum test

^b^ common odds ratio showing increase in odds per unit increase in level of the variable
