# Supplementary material for: Effectiveness of peer counseling and membership in breastfeeding support groups in promoting optimal breastfeeding behaviors in the Philippines
Source: Int Breastfeed J. 2021 Jul 12;16:53. doi: 10.1186/s13006-021-00400-5 (PMC8274007; doi:10.1186/s13006-021-00400-5)
Supplement: Supplementary file 4 — Additional file 4. Cross-tabulations and crude odds ratios of categorical variables with membership in breastfeeding support groups. Cross-tabulations and crude odds ratios of outcomes and probable confounders with membership in breastfeeding support groups as part of the assessment of potential confounding effects. [file 13006_2021_400_MOESM4_ESM.docx]

Additional File 4. Cross-tabulations and crude odds ratios of categorical variables with membership in breastfeeding support groups.

|  | Membership in breastfeeding support groups | | | p-value of χ^2^ | Crude ORs | p-value of crude OR |
| --- | --- | --- | --- | --- | --- | --- |
| Visit by a peer counselor during prenatal period | No | Yes | Missing | <0.01 |  |  |
| No | 1,443 (74.0) | 374 (26.0) | 0 (0.0) |  | 1  (baseline) |  |
| Yes | 230 (42.4) | 272 (57.6) | 0 (0.0) |  | 3.87  (2.74-5.46) | <0.01 |
| *Missing* | 10 (44.3) | 4 (29.6) | 10 (26.2) |  |  |  |
| Visit by a peer counselor after delivery |  |  |  | <0.01 |  |  |
| No | 1,421 (74.4) | 376 (25.6) | 0 (0.0) |  | 1  (baseline) |  |
| Yes | 259 (46.8) | 273 (53.2) | 0 (0.0) |  | 3.29  (2.38-4.55) | <0.01 |
| *Missing* | 3 (22.6) | 1 (18.2) | 10 (59.3) |  |  |  |
| Place of residence |  |  |  | 0.95 |  |  |
| Urban area | 1,535 (66.2) | 546 (33.5) | 10 (0.3) |  | 1  (baseline) |  |
| Rural area | 148 (66.7) | 104 (33.3) | 0 (0.0) |  | 0.99  (0.64-1.51) | 0.95 |
| Age of mothers in years |  |  |  | 0.01 |  |  |
| 15-19 | 135 (71.7) | 44 (28.3) | 0 (0.0) |  | 1  (baseline) |  |
| 20-24 | 473 (70.4) | 161 (29.6) | 0 (0.0) |  | 1.06  (0.67-1.69) | 0.80 |
| 25-29 | 469 (69.1) | 165 (30.9) | 0 (0.0) |  | 1.13  (0.74-1.73) | 0.57 |
| 30-34 | 362 (62.8) | 157 (37.1) | 2 (0.1) |  | 1.49  (0.94-2.36) | 0.09 |
| 35-39 | 165 (58.2) | 81 (41.8) | 0 (0.0) |  | 1.82  (1.04-3.17) | 0.04 |
| 40-50 | 76 (58.6) | 41 (39.4) | 3 (2.1) |  | 1.70  (1.03-2.80) | 0.04 |
| *Missing* | 3 (56.8) | 1 (13.5) | 5 (29.8) |  |  |  |
| Monthly income (PhP) |  |  |  | 0.08 |  |  |
| 0 – 3,800 | 343 (70.3) | 124 (29.7) | 2 (0.1) |  | 1  (baseline) |  |
| 3,801 – 5,999 | 281 (60.4) | 129 (39.4) | 1 (0.2) |  | 1.55  (1.05-2.28) | 0.03 |
| 6,000 – 8,999 | 378 (66.3) | 144 (33.5) | 2 (0.2) |  | 1.20  (0.84-1.71) | 0.32 |
| 9,000 – 15,999 | 341 (63.6) | 136 (36.1) | 3 (0.4) |  | 1.35  (0.93-1.94) | 0.11 |
| 16,000+ | 340 (69.9) | 117 (29.7) | 2 (0.4) |  | 1.01  (0.68-1.49) | 0.98 |
| Employment status of mother |  |  |  | 0.28 |  |  |
| Employed | 1,552 (66.0) | 608 (33.8) | 7 (0.2) |  | 1  (baseline) |  |
| Unemployed | 131 (69.8) | 42 (29.2) | 3 (1.1) |  | 0.82  (0.56-1.19) | 0.28 |
| Employment status of partner |  |  |  | 0.09 |  |  |
| Employed | 1,501 (66.2) | 589 (33.6) | 6 (0.2) |  | 1  (baseline) |  |
| Unemployed | 70 (75.1) | 15 (25.0) | 0 (0.0) |  | 0.65  (0.40-1.07) | 0.09 |
| *Missing/Not applicable* | 112 (63.0) | 46 (35.5) | 4 (1.5) |  |  |  |
| Marital status |  |  |  | 0.78 |  |  |
| Married/Living together | 1,567 (66.6) | 603 (33.3) | 6 (0.2) |  | 1  (baseline) |  |
| Never married/separated/  divorced/widowed | 114 (64.5) | 45 (34.0) | 4 (1.5) |  | 1.06  (0.72-1.55) | 0.78 |
| *Missing* | 2 (7.2) | 2 (92.8) | 0 (0.0) |  |  |  |
| Combined variable for civil status and employment status of partner |  |  |  | 0.28 |  |  |
| Single mother | 114 (64.5) | 45 (34.0) | 4 (1.5) |  | 1  (baseline) |  |
| Has employed partner/  Spouse | 1497 (66.3) | 587 (33.6) | 6 (0.2) |  | 0.96  (0.65-1.41) | 0.84 |
| Has unemployed  partner/spouse | 70 (75.1) | 15 (25.0)) | 0 (0.0) |  | 0.63  (0.36-1.11) | 0.11 |
| *Missing* | 2 (5.4) | 3 (94.6) | 0 (0.0) |  |  |  |
| Membership in 4Ps |  |  |  | <0.01 |  |  |
| No | 1,388 (70.5) | 457 (29.2) | 8 (0.3) |  | 1  (baseline) |  |
| Yes | 294 (52.6) | 191 (47.2) | 2 (0.3) |  | 2.17  (1.57-2.98) | <0.01 |
| *Missing* | 1 (26.7) | 2 (73.3) | 0 (0.0) |  |  |  |
| Prenatal care provider |  |  |  | 0.07 | 1  (baseline) |  |
| Doctor/Nurse/Midwife | 1,630 (65.8) | 641 (34.2) | 0 (0.0) |  | 0.32  (0.09-1.19) | 0.09 |
| None/Traditional Birth Attendant | 52 (85.8) | 9 (14.2) | 0 (0.0) |  |  |  |
| *Missing* | 1 (5.9) | 0 (0.0) | 10 (94.1) |  |  |  |
| Mode of delivery |  |  |  | 0.98 |  |  |
| Normal | 1,531 (66.2) | 599 (33.6) | 9 (0.3) |  | 1  (baseline) |  |
| Caesarean/other | 144 (66.1) | 50 (33.8) | 1 (0.1) |  | 1.01  (0.61-1.66) | 0.98 |
| *Missing* | 8 (91.0) | 1 (9.1) | 0 (0.0) |  |  |  |
| Birth attendant |  |  |  | 0.31 |  |  |
| Skilled | 1,290 (65.6) | 515 (34.4) | 0 (0.0) |  | 1  (baseline) |  |
| Traditional birth  attendant/none/self/  relatives/underboard  midwife | 381 (69.1) | 131 (30.9) | 0 (0.0) |  | 0.85  (0.63-1.16) | 0.31 |
| *Missing* | 12 (51.9) | 4 (20.1) | 10 (28.0) |  |  |  |
| Place of delivery |  |  |  | 0.30 |  |  |
| Home-based | 482 (67.5) | 185 (32.5) | 0 (0.0) |  | 1  (baseline) |  |
| Government healthcare facility | 1,038 (66.9) | 396 (33.1) | 0 (0.0) |  | 1.03  (0.73-1.42) | 0.88 |
| Private healthcare facility | 158 (59.5) | 67 (40.5) | 0 (0.0) |  | 1.41  (0.95-2.10) | 0.09 |
| *Missing* | 0 (0.0) | 1 (27.1) | 10 (72.9) |  |  |  |
| Gender of child |  |  |  | 0.30 |  |  |
| Boy | 835 (65.0) | 341 (34.7) | 6 (0.3) |  | 1  (baseline) |  |
| Girl | 848 (67.7) | 309 (32.1) | 4 (0.2) |  | 0.89  (0.71-1.12) | 0.32 |
| Initiation of breastfeeding |  |  |  | <0.01 |  |  |
| Late | 580 (72.3) | 181 (27.5) | 2 (0.1) |  | 1  (baseline) |  |
| Early | 1,032 (62.6) | 463 (37.1) | 5 (0.3) |  | 1.56  (1.17-2.08) | <0.01 |
| *Missing* | 71 (80.4) | 6 (18.4) | 3 (1.2) |  |  |  |
| Exclusive breastfeeding |  |  |  | <0.01 |  |  |
| No | 858 (70.5) | 292 (29.3) | 5 (0.3) |  | 1  (baseline) |  |
| Yes | 410 (58.1) | 210 (41.7) | 1 (0.2) |  | 1.73  (1.32-2.27) | <0.01 |
| *Missing* | 74 (73.6) | 12 (25.4) | 3 (1.0) |  |  |  |
| Month when prenatal care was first availed | | | | 0.61 | 0.93  (0.88-0.98) | <0.01 |
| Maternal knowledge score | | | | 0.36 | 1.14  (1.02-1.29) | 0.02 |
| Household size | | | | 0.02 | 1.04  (1.00-1.08) | 0.07 |
| Number of living older siblings | | | | <0.01 | 1.12  (1.07-1.17) | <0.01 |
