# Supplementary material for: Effectiveness of peer counseling and membership in breastfeeding support groups in promoting optimal breastfeeding behaviors in the Philippines
Source: Int Breastfeed J. 2021 Jul 12;16:53. doi: 10.1186/s13006-021-00400-5 (PMC8274007; doi:10.1186/s13006-021-00400-5)
Supplement: Supplementary file 5 — Additional file 5. Cross-tabulations and crude odds ratios of categorical variables with visit by a peer counselor after delivery. Cross-tabulations and crude odds ratios of outcomes and probable confounders with visit by a peer counselor after delivery as part of the assessment of potential confounding effects. [file 13006_2021_400_MOESM5_ESM.docx]

Additional File 5. Cross-tabulations and crude odds ratios of categorical variables with visit by a peer counselor after delivery.

|  | Visit by peer counselor after delivery | | | p-value | Crude OR | p-value of crude OR |
| --- | --- | --- | --- | --- | --- | --- |
| Membership in  breastfeeding support groups | No | Yes | Missing | <0.01 |  |  |
| No | 1,421  (79.8) | 259  (20.1) | 3  (0.2) |  | 1  (baseline) |  |
| Yes | 376  (54.5) | 273  (45.3) | 1  (0.2) |  | 3.29  (2.38-4.55) | <0.01 |
| *Missing* | 0  (0.0) | 0  (0.0) | 10  (100.0) |  |  |  |
| Peer counselor visit after delivery |  |  |  | <0.01 |  |  |
| No | 1,554  (80.3) | 261  (19.7) | 2  (0.01) |  | 1  (baseline) |  |
| Yes | 235  (43.2) | 267  (56.8) | 0  (0.0) |  | 5.37  (3.89-7.43) | <0.01 |
| *Missing* | 8  (35.7) | 4  (25.7) | 12  (38.6) |  |  |  |
| Place of residence |  |  |  | 0.01 |  |  |
| Urban area | 1,656  (74.4) | 422  (25.2) | 13  (0.5) |  | 1  (baseline) |  |
| Rural area | 141  (59.9) | 110  (39.8) | 1  (0.4) |  | 1.96  (1.21-3.18) | <0.01 |
| Age of mothers in years |  |  |  | 0.27 |  |  |
| 15-19 | 139  (68.9) | 40  (31.1) | 0  (0.0) |  | 1  (baseline) |  |
| 20-24 | 494  (71.8) | 139  (28.0) | 1  (0.2) |  | 0.86  (0.57-1.32) | 0.49 |
| 25-29 | 495  (72.9) | 138  (26.9) | 1  (0.2) |  | 0.82  (0.50-1.33) | 0.42 |
| 30-34 | 398  (73.2) | 120  (26.7) | 3  (0.1) |  | 0.81  (0.50-1.31) | 0.38 |
| 35-39 | 178  (63.3) | 68  (36.7) | 0  (0.0) |  | 1.28  (0.77-2.13) | 0.33 |
| 40-50 | 91  (71.2) | 25  (25.4) | 4  (3.5) |  | 0.79  (0.39-1.60) | 0.51 |
| *Missing* | 2  (41.9) | 2  (28.4) | 5  (29.8) |  |  |  |
| Monthly income (PhP) |  |  |  | 0.48 |  |  |
| 0 – 3,800 | 360  (74.0) | 105  (25.3) | 4  (0.7) |  | 1  (baseline) |  |
| 3,801 – 5,999 | 320  (70.5) | 89  (29.0) | 2  (0.5) |  | 1.20  (0.83-1.75) | 0.33 |
| 6,000 – 8,999 | 396  (67.6) | 126  (32.2) | 2  (0.2) |  | 1.39  (0.94-2.07) | 0.10 |
| 9,000 – 15,999 | 362  (72.0) | 114  (27.6) | 4  (0.4) |  | 1.12  (0.80-1.58) | 0.51 |
| 16,000+ | 359  (71.3) | 98  (28.3) | 2  (0.4) |  | 1.16  (0.79-1.70) | 0.44 |
| Employment status of mother |  |  |  | 0.50 |  |  |
| Employed | 1,660  (71.0) | 498  (28.7) | 9  (0.2) |  | 1  (baseline) |  |
| Unemployed | 137  (72.3) | 34  (25.1) | 5  (2.6) |  | 0.86  (0.55-1.34) | 0.50 |
| Employment status of partner |  |  |  | 0.31 |  |  |
| Employed | 1,600  (70.5) | 488  (29.3) | 8  (0.2) |  | 1  (baseline) |  |
| Unemployed | 69  (76.5) | 15  (21.5) | 1  (2.0) |  | 0.67  (0.31-1.46) | 0.31 |
| *Missing/Not applicable* | 128  (75.7) | 29  (22.2) | 5  (2.1) |  |  |  |
| Civil status |  |  |  | 0.16 |  |  |
| Married/Living together | 1,666  (70.8) | 501  (28.9) | 9  (0.3) |  | 1  (baseline) |  |
| Never married/separated/  divorced/widowed | 129  (76.2) | 29  (21.7) | 5  (2.0) |  | 0.70  (0.42-1.16) | 0.16 |
| *Missing* | 2  (7.2) | 2  (92.8) | 0  (0.0) |  |  |  |
| Combined variable for civil status and employment status of partner |  |  |  | 0.23 |  |  |
| Single mother | 129  (76.2) | 29  (21.7) | 5  (2.0) |  | 1  (baseline) |  |
| Has employed partner/  spouse | 1,596  (70.6) | 486  (29.2) | 8  (2.2) |  | 1.45  (0.87-2.43) | 0.15 |
| Has unemployed  partner/spouse | 69  (76.5) | 15  (21.5) | 1  (2.0) |  | 0.98  (0.42-2.28) | 0.97 |
| *Missing* | 3  (29.7) | 2  (70.3) | 0  (0.0) |  |  |  |
| Membership in 4Ps |  |  |  | 0.16 |  |  |
| No | 1,437  (72.2) | 406  (27.5) | 10  (0.3) |  | 1  (baseline) |  |
| Yes | 358  (67.7) | 125  (31.5) | 4  (0.8) |  | 1.22  (0.92-1.61) |  |
| *Missing* | 2  (73.3) | 1  (26.7) | 0  (0.0) |  |  |  |
| Prenatal care provider |  |  |  | 0.02 |  |  |
| Doctor/Nurse/Midwife | 1,743  (70.8) | 524  (29.0) | 4  (0.2) |  | 1  (baseline) |  |
| None/Traditional Birth Attendant | 53  (84.9) | 8  (15.1) | 0  (0.0) |  | 0.43  (0.21-0.91) | 0.03 |
| *Missing* | 1  (6.0) | 0  (0.0) | 10  (94.0) |  |  |  |
| Mode of delivery |  |  |  | 0.97 |  |  |
| Normal | 1,640  (71.0) | 486  (28.5) | 13  (0.5) |  | 1  (baseline) |  |
| Caesarean/other | 149  (71.4) | 45  (28.5) | 1  (0.1) |  | 0.99  (0.69-1.43) | 0.97 |
| *Missing* | 8  (84.5) | 1  (15.5) | 0  (0.0) |  |  |  |
| Birth attendant |  |  |  | 0.55 |  |  |
| Skilled | 1,392  (70.6) | 410  (29.2) | 3  (0.2) |  | 1  (baseline) |  |
| Traditional birth  attendant/none/self/  relatives/underboard  midwife | 392  (73.2) | 119  (26.7) | 1  (0.2) |  | 0.88  (0.57-1.35) | 0.54 |
| *Missing* | 13  (59.7) | 3  (12.3) | 10  (28.0) |  |  |  |
| Place of delivery |  |  |  | 0.74 |  |  |
| Home-based | 502  (72.4) | 163  (27.2) | 2  (0.4) |  | 1  (baseline) |  |
| Government healthcare facility | 1,109  (70.5) | 323  (29.4) | 2  (0.1) |  | 1.11  (0.79-1.56) | 0.53 |
| Private healthcare facility | 179  (70..8) | 46  (29.2) | 0  (0.0) |  | 1.10  (0.70-1.72) | 0.67 |
| *Missing* | 7  (70.0) | 0  (0.0) | 10  (30.0) |  |  |  |
| Gender of child |  |  |  | 0.26 |  |  |
| Boy | 892  (69.8) | 283  (29.7) | 7  (0.5) |  | 1  (baseline) |  |
| Girl | 905  (72.5) | 249  (27.1) | 7  (0.4) |  | 0.88  (0.70-1.10) | 0.26 |
| Initiation of breastfeeding |  |  |  | <0.01 |  |  |
| Late | 614  (77.2) | 146  (22.6) | 3  (2.6) |  | 1  (baseline) |  |
| Early | 1,117  (67.5) | 375  (32.1) | 8  (0.1) |  | 1.62  (1.18-2.24) |  |
| *Missing* | 66  (82.6) | 11  (16.2) | 3  (1.2) |  |  |  |
| Exclusive breastfeeding |  |  |  | 0.05 |  |  |
| No | 890 (71.9) | 258 (27.6) | 7 (0.1) |  | 1  (baseline) |  |
| Yes | 456 (66.9) | 163 (32.9) | 2 (0.1) |  | 1.28  (0.97-1.70) | 0.09 |
| *Missing* | 73 (82.6) | 13 (16.4) | 3 (1.0) |  |  |  |
| Months when prenatal care was first availed | | | | 0.36 | 0.98  (0.91-1.06) | 0.62 |
| Maternal knowledge score | | | | 0.90 | 1.16  (1.01-1.32) | 0.04 |
| Household size | | | | 0.34 | 1.01  (0.98-1.05) | 0.79 |
| Number of living older siblings | | | | 0.19 | 1.03  (0.95-1.11) | 0.45 |

^a^ p-value from Wilcoxon rank-sum test

^b^ common odds ratio showing increase in odds per unit increase in level of the variable
